# Attention Module Magnetic Flux Leakage Linked Deep Residual Network for Pipeline In-Line Inspection

**DOI:** 10.3390/s22062230

**Published:** 2022-03-14

**Authors:** Shucong Liu, Hongjun Wang, Rui Li

**Affiliations:** 1 School of Mechanical and Electrical Engineering, Beijing Information Science and Technology University, Beijing 100192, China; liushucong@bistu.edu.cn; 2 College of Mechanical Engineering and Applied Electronics Technology, Beijing University of Technology, Beijing 100124, China; 3 PipeChina Northern Company, Langfang 065000, China; kjlirui@petrochina.com.cn

**Keywords:** long-distance oil and gas pipeline, MFL in-line inspection, feature identification, residual network, convolutional block attention module

## Abstract

Pipeline operational safety is the foundation of the pipeline industry. Inspection and evaluation of defects is an important means of ensuring the safe operation of pipelines. In-line inspection of Magnetic Flux Leakage (MFL) can be used to identify and analyze potential defects. For pipeline MFL identification with inspecting in long distance, there exists the issues of low identification efficiency, misjudgment and leakage judgment. To solve these problems, a pipeline MFL inspection signal identification method based on improved deep residual convolutional neural network and attention module is proposed. A improved deep residual network based on the VGG16 convolution neural network is constructed to automatically learn the features from the MFL image signals and perform the identification of pipeline features and defects. The attention modules are introduced to reduce the influence of noises and compound features on the identification results in the process of in-line inspection. The actual pipeline in-line inspection experimental results show that the proposed method can accurately classify the MFL in-line inspection image signals and effectively reduce the influence of noises on the feature identification results with an average classification accuracy of 97.7%. This method can effectively improve identification accuracy and efficiency of the pipeline MFL in-line inspection.

## 1. Introduction

Oil and gas pipeline transportation is one of the five major transportation industries, along with railway, highway, aviation, and water transportation [1]. Since long-distance oil and gas pipelines have merits of low transportation cost, stable transportation capacity, and uninterrupted transportation, they have become the most important means of large-scale oil and gas transportation in the world [2]. Oil and gas pipelines have been built in land, sea, mountains, and other geographical environments around the world on a large scale, being the lifeblood of modern industry and national economy [3]. The most basic requirement for pipeline operation is safety and stability. With the increase of service age and the change of geological conditions, the pipeline is vulnerable to welds, oil and gas corrosion, and man-made damage, resulting in pipeline failure. If it is not discovered and performed effective maintenance in time, oil and gas leakage or explosion accidents will occur which endanger people’s lives and result in environmental pollution and serious social impacts [4]. Therefore, it is necessary to conduct regular corrosion inspections and pipeline health conditions management according to the inspection results. Many years of pipeline operation practice at home and abroad show that the whole-life inspection and evaluation of pipelines is one of the most critical methods to ensure the integrity and reliability of pipelines. In-line inspection is the most important technical means for the detection or perception of the condition of oil and gas pipelines [5].

In-line inspection technology is a pipeline inspection method using an intelligent in-line inspection tool. This tool is installed in different types of nondestructive detection equipment or related sensors on the vessel according to the type of pipeline defects, of which the pig for cleaning dirt or impurities is refitted into an intelligent in-line tool integrating data acquisition, signal processing, storage control, and other functions [6,7]. This intelligent in-line inspection tool driven by the medium in the pipeline collects the information of the entire pipeline through various sensors and electronic systems. These data are used to determine the type and size of pipeline defects through later offline analysis of inspection data to provide an effective method to guarantee the safe operation of the pipeline [8]. Developed industrial countries have attached great attention to the research and development of oil and gas pipeline inspection technologies. After decades of development, a series of mature technologies and equipment have been developed both at home and abroad, and have been used for thousands of kilometers of oil and gas pipeline inspection, and good results have been achieved providing a huge safety guarantee for oil and gas pipeline operators [9].

MFL is the most commonly used in-line inspection method for determining the metal loss in oil and gas pipelines. MFL has the advantages of wide application range, such as without couplant, high detection reliability and accuracy, and can effectively detect metal loss caused by corrosion or scratches and external metal objects [10]. However, for long distance inspection of oil and gas pipelines, some problems exist, such as low efficiency, misjudgment, and omission of subsequent signal identification and analysis. Therefore, the focus of current research is to identify and discriminate MFL inspection signals with high efficiency and reliability. With the development of MFL in-line inspection technology, scholars have conducted relevant research on signal identification and analysis. Liu [11] designed a defect identification algorithm based on a random forest, which was used to recognize various types of defects in MFL inner-detection data. Chen [12] proposed an iterative neural network to reconstruct three-dimensional defect profiles from three-axial MFL signals in pipeline inspection, which was robust even in the presence of reasonable noise. MR Kandroodi [13] proposed an axial flux detection algorithm for defect detection based on image processing approaches and morphological methods, which was validated through examinations of simulated defects and real experimental MFL data. Sorabh [14] proposed a three-dimensional finite element model and static simulation that studied the dependency of the characteristic defect dimensions and the leakage flux signal. In recent years, with the development of artificial intelligence, deep-learning methods have been applied in the field of pipeline MFL inspection. After the preprocessed data are input into the deep learning model, the model automatically performs feature extraction for identification and classification, which achieves good identification results. Wang [15] proposed a pipeline magnetic flux leakage image detection algorithm based on a multi-scale SSD network, which automatically identified the location of the girth weld, spiral weld, and defects in the magnetic flux leakage data. Yang [16] proposed a method for magnetic flux leakage image classification based on sparse self-coding, which had good feature extraction and generalization abilities. Lu [17] proposed a novel visual transformation CNN to estimate the defect size in specimens from the MFL.

However, the pipeline MFL in-line tool is prone to being affected by impact and vibration during operation, resulting in high signal background noises [18]. The tool sensors are often lifted off owing to the continuous touching of the girth and spiral weld, resulting in a decrease in signal strength. In addition, because of the long inspection distance of the pipeline, the features or defects of the pipeline are compound, and the superposition of different features or defects will affect the identification accuracy. Deep learning methods have become a hotspot in the research of signal identification and fault diagnosis in recent years. At present, researches of pipeline MFL signal identification method based on deep learning frameworks have obtained results [19,20]. The complexity of pipeline MFL signals brings limitation on identification accuracy by using traditional methods.

Therefore, to further improve the identification accuracy for the complex pipeline MFL signals, a pipeline MFL inspection feature identification model based on an improved deep residual convolutional neural network is proposed. The experiment results showed that the proposed method not only automatically learns the features from the MFL inspection images and performs the classification and identification of pipeline features and defects such as welds, tees, flanges, and corrosion, but also solves the problems of the great influence of noise, compound features, and other factors on the feature identification results in the process of in-line inspection. Compared with other methods, the proposed method effectively improves the classification of pipeline features, and provides an effective method for pipeline features identification.

The main contributions of this paper are as follows:

(1) An MFL in-line inspection method based on attention module and convolution residual modules is proposed for oil and gas pipeline, which effectively improve the pipeline features inspection accuracy and efficiency.
(2) Aiming at the influence of the complex operating environment, high noises, composite defects to MFL in-line inspection of oil and gas pipelines, attention module composed of channel attention and spatial attention are designed to fully extract MFL image feature information.
(3) To solve the problem of gradient dispersion caused by the increase of the number of network layers, an improved residual convolutional neural network is constructed to reduce the error of the deep network as well as the amount of calculation parameters, and effectively improve the training efficiency.

The remaining parts of the paper are as follows. Section 2 provides the methods which include the basic CNN and the MFL in-line inspection technology for pipelines and the proposed method for the pipeline MFL signal identification. Section 3 shows the experiment and result analysis. Section 4 is the conclusion.

## 2. Methods

### 2.1. Related Work

#### 2.1.1. Convolutional Neural Network (CNN)

**Figure 1:**
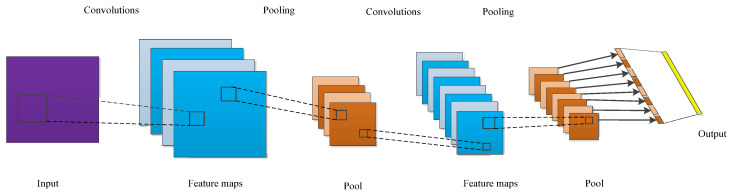
Structure of convolutional neural network.

A CNN is a typical feed forward neural network and one of the most popular deep learning algorithms. It can learn the mapping relationship between the input and output using a large amount of data. As long as the known convolutional network model is trained with sample data, it can obtain a network model with good performance to extract the local features of the images. This network model has been widely applied in image identification and classification, face recognition, audio retrieval, natural language processing, visual tracking, and other fields [21,22]. Yuan proposed an intelligent fault diagnosis method for rolling bearings based on wavelet time-frequency map and CNN. The improved CNN with strong generalization, feature extraction and identification can effectively identify the fault types of rolling bearings [23]. D Neupane presented the method that detects bearing failures using the continuous wavelet transform and classifies them using a switchable normalization-based convolutional neural network [24]. To solve the problem of a lack of labeled samples with the same distribution in real industry, J He proposed a deep transfer learning method based on special 1D-CNN for rolling bearing fault diagnosis [25]. Zheng designed a new fault diagnosis method using deformable CNN, deep long short-term memory and transfer learning strategies. The method can be used for insufficient labeled vibration data and inevitable dynamic changes of multiple working conditions [26]. The CNN is composed of an input layer, convolutional layer, pooling layer, full connection layer, and output layer, as shown in Figure 1. The input layer realizes the input of the original image dataset, the convolutional layer obtains the feature map through convolution calculation, and the pooling layer performs a downsampling operation on the feature map to reduce the data dimension. The number of convolutional layers and downsampling layers are often determined according to the actual situation. The full connection layer mainly realizes data mapping [27].

A convolutional layer is used to extract the features of the input images. The image with pixel size of M×N is represented as P=f(x,y), f(x,y) is the gray value of the point in the *x*-th row and *y*-th column of the image *P*. The convolution kernel is represented as K(x,y), with a size of a×b. C(s,t) is the convolution operation matrix of image *P* and convolution kernel *K*.

(1) $$C\left(s,t\right)=f\left(x,y\right)\times k\left(x,y\right)=\sum_{x=1}^{a}\sum_{y=1}^{b}k\left(x,y\right)f\left(s+x-1,t+y-1\right)$$

where 1≤s≤M−a+1, 1≤t≤N−b+1. The convolution operation continuously moves the convolution kernel on the image matrix and convolutes it with the corresponding segment to form a new image matrix. The primary function of the convolutional layer is feature extraction. Through a convolution operation, signal features can be enhanced and noises can be reduced.

The pooling layer, also known as the down-sampling layer, divides the input feature maps into many non-overlapping rectangular regions. The maximum value extraction of each region is called max pooling, and the average value extraction of each region is called average pooling. The essence of pooling is the dimensionality reduction. Pooling operations downsample the feature image obtained by the convolution layer through the downsampling layer, which can reduce the amount of calculation and avoid over fitting.

Convolution operations only have the ability of linear mapping, and cannot meet the needs of feature extraction. A nonlinear function called the activation function is often added after the convolution operation. Common activation functions include the Sigmoid, tanh, ReLU, and Softmax functions. Among these, the ReLU function is the most widely used. The main advantages of the ReLU function are: ① when *x* > 0, the gradient is 1, there is no gradient saturation problem, and the convergence speed is fast; and ② when *x* < 0, the output is 0, which increases the network sparsity and improves the generalization ability.

The full connection layer is located behind the convolutional and pooling layers to organize and synthesize the extracted features. The input of the first full connection layer is the feature maps obtained by feature extraction through convolution and pooling. The last output layer is a classifier that can classify signals. Softmax is commonly used as the classifier.

Before training the CNN, the parameters in the network model were initialized. Then, forward propagation was performed. Through a series of operations, such as convolution, pooling, and activation functions, the hidden layer in the network structure is used for feature extraction and mapping. The high-level semantic information is extracted from the input image layer by layer, and the image category information is obtained through the full connection layer. In the network training stage, the error between the predicted value and the real value is calculated and fed forward layer by layer from the last layer using the back propagation algorithm, which is supervised by the network model to update the parameters of each layer to reduce the error. Then, the feed forward operation and back propagation are repeated until the network model converges. The classification and identification ability of a CNN mainly depends on the learning ability of the network for the images, that is, the feature extraction ability of the convolution kernel for images. The quality of the convolution kernel is determined by its internal weight, and the selection of the weight in the network is very important.

#### 2.1.2. VGG16 Deep Convolutional Neural Network

The VGG16 network is a deep network model developed by the Computer Vision Team of Oxford University and researchers of Google DeepMind in 2014. The network has 16 training parameters, with a simple network structure and excellent generalization performance when transferred to other image data [28,29]. The VGG16 model exhibited excellent performance in classification and achieved the best effect on multiple datasets at that time. At present, the VGG16 network is still widely used as a feature extraction network to extract features, mainly including the input, convolutional, pooling, full connection, and Softmax layers. The input images are preprocessed images of the same size, and the features are extracted by the convolutional layer to obtain a certain number of feature maps. Then, the feature maps are input into the pooling layer for downsampling to generalize the feature maps, and the ReLU function is used as the activation function behind each convolutional layer, which makes the function nonlinear. Qian used the VGG-16 convolutional neural network as the core network structure, and constructed an intelligent identification model of rice pests based on VGG16 convolutional neural network according to the individual characteristics of rice pests and natural scenes [30]. J Duan proposed a fully automatic online monitoring method incorporating a K-means clustering-based haze judgment module, a lightweight U-net segmentation model with the fusion of none-weight VGG16 features. This method can accurately segment the pellets from both hazy and haze-free images with the help of the haze judgment module [31]. Z Omiotek presented a method combining flame image processing with a deep convolutional neural network that ensures high accuracy of identifying undesired combustion states based on the pretrained VGG16 model [32]. In order to solve the problems of few images, a lot of manual annotation and low efficiency in the process of chip image classification, Ma proposed a VGG16 network chip image classification method based on transfer learning. It can automatically learn image features in the process and effectively reduce the cost of manual annotation [33].

**Figure 2:**
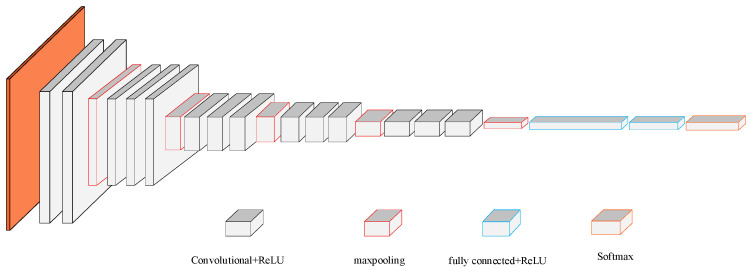
VGG16 network model.

The network structure diagram of VGG16 is shown in Figure 2. The orange part represents the input signal images. If the size of the input signal image is 224 × 224 × 3, 3 indicates that the image is colored, and 224 is the pixel value of the image. Black cubes are convolutional layers that are mainly used to extract features. The red cubes are pooling layers, which are mainly used to retain the main features of the images.

Firstly, the image is convoluted twice with 64 convolution kernels of 3 × 3 in size and 1 in stride, and the size after convolution operation becomes 224 × 224 × 64, then a max pooling calculation is performed, with the pooling unit size of 2 × 2 and the stride of 2; Next, two convolution operations of 128 convolution kernels and one max pooling calculation are carried out for the image, and the size becomes 112 × 112 × 128, during which the pooling unit size is 2 × 2, the stride is 2 and the output is 56 × 56 × 128. Then, three convolution operations of 256 convolution kernels and one max pooling calculation are carried out for the image, during which the convolution kernel size is 3 × 3 with the stride of 1, the pooling unit size is 2 × 2 with the stride of 2, the output size after convolution calculation is 56 × 56 × 256, and the output size after pooling calculation is 28 × 28 × 256. Then, the calculation of three convolutional layers are performed for the image, with 512 convolution kernels of the same size and stride as that of the previous convolutional layer, and the output size is 28 × 28 × 512. One max pooling calculation is performed, with the same pooling unit size and stride as that of the previous pooling layer, and the output size is 14 × 14 × 512. Next, the calculations of three convolutional layers are performed for the image, with 512 convolution kernels of the same size and stride as that of the previous convolutional layer, and the output size is 14 × 14 × 512. Then, the max pooling calculation is performed for the image, with the same pooling unit size and stride as that of the previous pooling layer, and the output size is 7 × 7 × 512. After completing the above calculation, three fully connected layer operations are performed on the image, where the blue cubes are the fully connected layers, and the final output is the category probability of the image. Each convolutional layer is connected to a ReLU activation function.

Although VGG16 has a simple structure, it contains a large number of weights, resulting in a long training time, difficulty in parameter adjustment, and requirement of large storage memory, which is not conducive to MFL in-line inspection under complex working conditions. On this basis, simply increasing the network depth for accuracy improvement can easily cause gradient explosion or gradient disappearance, and large amount of computing. Therefore, the network needs to be improved.

### 2.2. Proposed Attention Module MFL In-Line Inspection Technology for Pipeline

#### 2.2.1. MFL In-Line Inspection Tool

**Figure 3:**
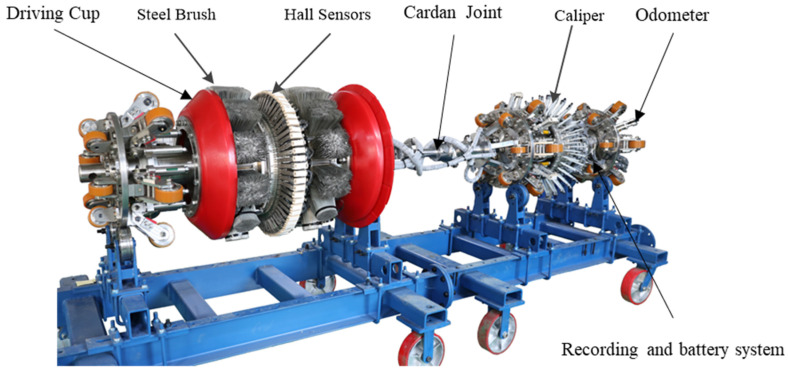
MFL in-line inspection tool.

As shown in Figure 3, the MFL in-line inspection tool is often composed of a driving cup, magnetic flux leakage unit, caliper, recording and battery system, and odometers, which are connected by a Cardan joint in the middle. The MFL unit consists of a yoke, a permanent magnet, several steel brushes and Hall sensors. It is a central part of the inspection tool and can collect signals of the magnetic field and MFL on the magnetized pipeline wall. Different sizes of in-line inspection tool have different numbers of Hall sensors covering the circumference of the pipeline. When there are more Hall sensors, the collected signals are clearer. Driven by the medium transported in the pipeline, the tool can move forward inside the pipeline under the pressure difference in front of and behind the driving cup to identify and inspect the corrosion defects inside and outside the pipeline, wall thickness variation, weld defects, and pipeline features. Additionally, the tool can provide information on defects, including area, depth, orientation, and position.

#### 2.2.2. Principle and Disturbance for Pipeline MFL In-Line Inspection

**Figure 4:**
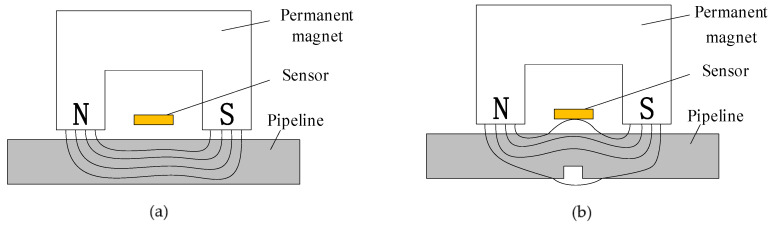
Principles of magnetic flux leakage inspection [34]. (**a**) Distribution of magnetic lines of force without defect, (**b**) Distribution of magnetic lines of force with defect.

The technical principle of MFL in-line inspection is to judge the severity of defects in an inspected workpiece by measuring the MFL on the surface of a magnetized ferromagnetic material. After a ferromagnetic material is magnetized by an externally applied magnetic field, if it is continuous and uniform, the magnetic lines of force in the material will be constrained inside the material and will not penetrate the surface of the material, as shown in Figure 4a [34]. However, if there are any defects on the surface or inside the ferromagnetic material, there is low magnetic permeability and high magnetic reluctance at the position of the defect. The magnetic lines of force first pass through the area with a lower magnetic reluctance. When the magnetic induction intensity inside the material is high or the defect is large, the material near the defect cannot bear a higher magnetic flux. Thus, a portion of the magnetic flux flows out of the workpiece from the position of the defect, passes through the workpiece above the defect, and then reenters the workpiece. In this manner, a magnetic leakage field is formed outside the workpiece, as shown in Figure 4b [34]. The total magnetic flux through the cross-sectional area of the steel plate is [35]:

(2) $$\phi =B_{1}S_{1}$$

where $S_{1}$ is the area of pipeline wall without defects. According to the principle of continuous boundary flux, the flux $B_{o}$ on the outer surface of the workpiece is:

(3) $$B_{o}=B_{1}\frac{\mu_{s}}{\mu_{1}}$$

where $B_{1}$ is the magnetic induction intensity in the workpiece without defects, $\mu_{s}$ is the relative permeability of air, $\mu_{1}$ is the relative permeability of the workpiece, $\mu_{s}<\mu_{1}$. When the in-line inspection tool passes through the pipeline defect, the $B_{1}$ changes to $B_{2}$. Some magnetic induction lines overflow the pipeline wall surface and produce magnetic flux leakage. The magnetic sensor is used to collect the magnetic leakage field and convert it into electrical signals. Electrical signals were calculated to determine the condition of the defects.

The MFL signal of the pipeline contains all the information for the inspected pipeline. Through the analysis of the MFL, the size and position of pipeline welds, defects, and other features can be obtained. The MFL is a three-dimensional vector, which is divided into axial component along the pipeline forward direction, radial component perpendicular to the pipeline wall and circumferential component along the pipeline circumferential direction.

After MFL in-line inspection, the actual detected values of each channel in the sensors of the tool are imaged using line tracing points, and the curved image is generated first. Generally, the tool samples according to a certain sampling spacing in the forward direction of a pipeline. The smaller the sampling point spacing and the more circumferential the channels, the higher the accuracy of the tool and the more thorough the inspection of the pipeline. In the curved image, the abscissa represents the travel distance of the tool, and the ordinate represents the magnitude of the MFL collected by each channel.

**Figure 5:**
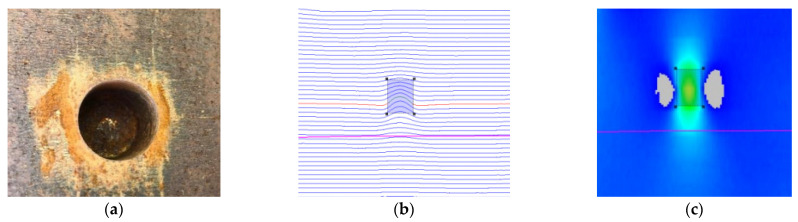
Pipeline defect and MFL image. (**a**) Real defect. (**b**) MFL curves for defect. (**c**) Pseudo-color image.

However, for image classification based on deep learning, the contrast of the curved image is poor, and the feature extraction of the network is difficult. Therefore, pseudo-color image processing technology can be used to process curved images to improve their resolution. Pipeline MFL pseudo-color image is used to directly map the converted MFL signal data to the RGB space, encode the three components, and finally fuse the three components into a pseudo-color image according to different proportions to obtain a complete MFL pseudo-color image [36,37]. MFL pseudo-color image can be expressed as:

(4) $$I(x,y)=\left(\begin{array}{l} i(0,0)\;i(0,1)\;i(0,2)\;\ldots \;i(0,N)\\ i(1,0)\;i(1,1)\;i(1,2)\;\ldots \;i(1,N)\\ \;\ldots \;\ldots \;\ldots \;\\ i(M,0)\;i(M,1)\;i(M,2)\;\ldots \;i(M,N) \end{array}\right)$$

where the i(x,y) represents the pseudo-color image pixel corresponding to the actual MFL inspection value. M is the number of sensor channels for MFL in-line inspection tool. The more sensors used, the greater the number of signal channels, and the pseudo-color image of MFL is clearer. N is the sampling spacing in the forward direction of a pipeline. The smaller the sampling spacing, the clearer the pseudo-color image of MFL. Figure 5 shows a comparison of the real defect, MFL curve, and pseudo-color image.

**Figure 6:**
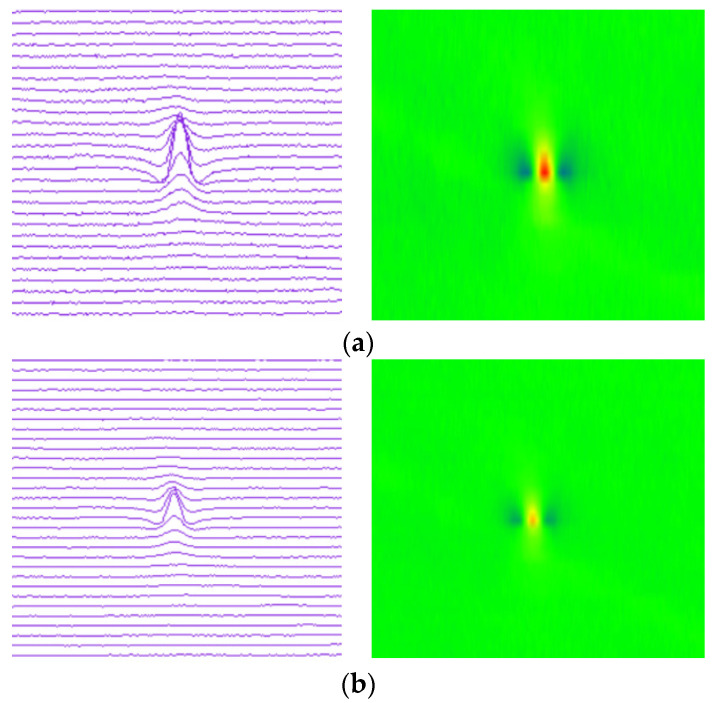

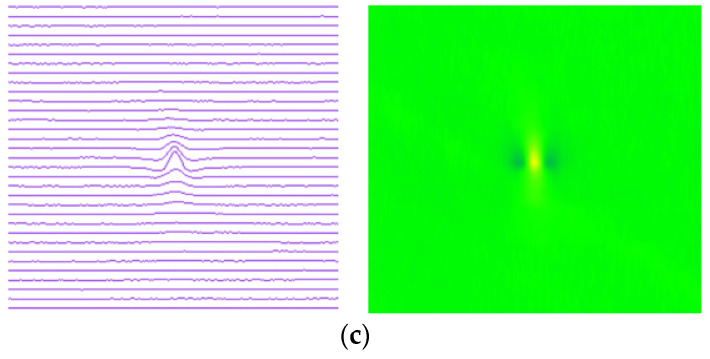
MFL signal of defect with different sensor lift-off. (**a**) Sensor lift-off is 0 mm. (**b**) Sensor lift-off is 1 mm. (**c**) Sensor lift-off is 2 mm.

In the process of in-line inspection, the MFL signal will be affected by pipeline internal environment, impact vibration, and other factors, such as the corrosion defects, welds, wax, or waste in the pipeline. These factors will lead to the lift-off of the sensors. The lift-off sensors will produce interference signals from the dynamic lift-off, thus affecting the inspection results. Figure 6 shows the MFL signals of the sensor with different lift-off values at the same corrosion defect. The lift-off of the sensors are 0 mm, 1 mm, and 2 mm, respectively. When the sensor is close to the pipeline wall (with 0 mm lift-off), the inspection signal amplitude is the largest and the signal is clear; when the sensor is lifted away by 1 mm and 2 mm respectively, it can be seen from the figure that the leakage magnetic field strength detected by the sensor becomes weaker. If the sensor is lifted-off to a certain distance, it will cause inspection failure. In addition, pipeline defects or features do not exist singly. Different defects or features will also compound together. As the input of deep neural network, the pseudo-color image of magnetic flux leakage detection is also affected by the lift-off value or compound signals, which makes feature extraction more difficult.

#### 2.2.3. Convolutional Attention Module for MFL In-Line Inspection

The internal noise, composite defects or features of the pipeline will increase the differences between similar samples and reduce the differences between different types of samples, resulting in the reduction of the accuracy of feature identification of pipeline MFL inspection. Therefore, it is necessary to improve the deep convolution network and propose a new mechanism to enhance the signal feature extraction ability of the network. The attention model was derived from a human visual attention model. While processing data, the visual system quickly focuses on the target areas that need to be focused by scanning the global scene, and allocates limited computing resources to these key parts. This mechanism can greatly reduce the amount of data to be processed, ignore unimportant information, and provide more manageable and relevant information for higher-level perceptual reasoning and complex visual processing. It is one of the core technologies in deep learning worthy of attention and in-depth understanding. The convolutional block attention module (CBAM) is an attention module combining spatial with channel information. CBAM adopts max-pooling and average-pooling to generate weights through the channel and spatial dimensions [38,39]. Adding the attention mechanism module can further extract the interested small defects target area from the background, thus the network can better learn small target defects. At the same time, the interference of the background to the target is suppressed, thus improving the learning ability of the network to the detailed features of small targets and enhance the ability of feature learning.

The method introduces the attention mechanism and designs the spatial attention module (Spatial_AM) and the channel attention module (Channel_AM). In Channel_AM, the property of maximum pooling is used to capture the inter class information between MFL image pixels, and the average pooling is used to capture the intra class information between pixels. These two information as weights are applied to the original feature map as attention to assist feature extraction. The module is connected between the feature map extraction module and the feature map decoding module. At the same time, the Spatial_AM is also designed. By using the spatial attention mechanism composed of global pooling, convolution, and activation function, the semantic information extraction is further refined, and the information is multiplied with the original feature map as a weight.

• Channel attention module

**Figure 7:**
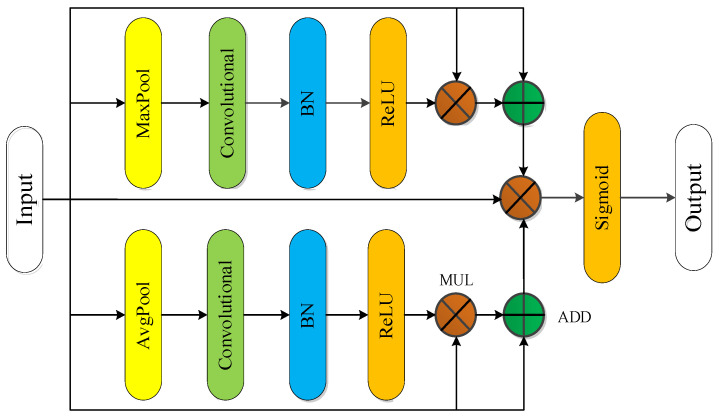
Channel attention module.

When extracting features in the channel dimension, average pooling and max pooling are considered simultaneously. Figure 7 shows the design scheme of the channel attention module.

The module consists of convolution, batch regularization, and an activation function, which can extract mixed information by integrating channel and semantic information. Then, the average pooling module, convolution and activation function ReLU are adopted to process the features, where ADD is the addition operation and MUL is the multiplication operation, to obtain the function $X_{avg}^{c}$; at the same time, the max pooling module, convolution and activation function ReLU are used in parallel with the average pooling module for another feature extraction to obtain the function $X_{Max}^{c}$. The designed attention feature map has features of both average pooling and max pooling. The attention feature is multiplied with the input feature map and superimposed with the input feature to as the weight to influence the input feature map. Finally, a structure similar to the jump connection was used to reduce the negative impact of the attention module on the input feature map, and the Sigmoid activation function was used to output the final feature map [40,41]. The calculation process is as follows.

(5) $$\begin{array}{ll} M_{c}(X) & =\sigma \left(f\left(AvgPool(X);MaxPool(X)\right)\right)\\ & =\sigma \left(f\left(X_{avg}^{c};X_{Max}^{c}\right)\right) \end{array}$$

The designed module can not only efficiently guide the acquisition of intraclass information through the average pooling operation, but also extracts more edge information through the max pooling operation, which can efficiently improve the acquisition of feature information.

• Spatial attention module

**Figure 8:**
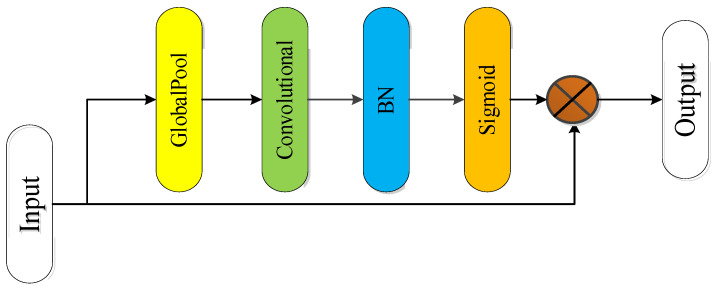
Spatial attention module.

By using the spatial attention module, useful information in the input image can be focused on. Figure 8 shows the designed spatial attention module, which focuses on the spatial or semantic feature information in the feature map [42]. By use of global average pooling, the length and width of the feature map are compressed into one, leaving only the spatial information. Then, the convolutional layer is used to learn the association between spatial information and classification information (semantic information), and batch regularization and activation function Sigmoid are used to transform this association into nonlinear change [43]. To avoid excessive loss of feature information caused by pooling, it was multiplied by the feature map without average pooling. The result of multiplication is input to the next module as the weight to influence the input feature map, so as to complete the task of refining semantic information.

### 2.3. Intelligent Identification of MFL In-Line Detection Signal of Oil and Gas Pipeline Based on Deep Residual Convolutional Neural Network

In the actual process of in-line inspection, the signal-to-noise ratio of the inspection signals is low because of the influence of the pipeline internal environment, impact vibration, and other factors. Additionally, compound defects or features will increase the differences between samples of the same category and reduce the differences between samples of different categories, thus reducing the accuracy of feature identification. In this study, a new deep neural network model is effectively constructed by constructing a residual network model and designing an attention model to enhance the feature learning ability of MFL in-line inspection signals to improve the identification accuracy of pipeline MFL feature signals under different conditions.

#### 2.3.1. Residual Network Design

Deep convolutional networks have an efficient feature extraction ability and can form more abstract high-dimensional features by combining low-dimensional features. It is generally believed that the more layers of the network, the richer the features that can be extracted, and the stronger the representation ability. However, as the number of network layers increases, the gradient of back propagation will become unstable and become extremely large or small, which makes the model difficult to train and converge, resulting in the gradient dispersion or explosion. On the other hand, with the network layers increases, the network performance tends to be saturated or even declines sharply, and the network information cannot be transmitted effectively, resulting in network degradation [44,45].

**Figure 9:**
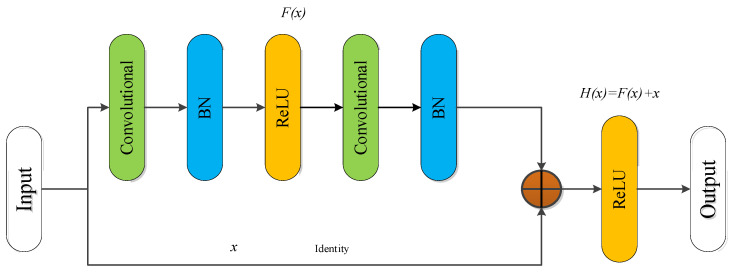
Identity residual module.

To solve the problems of gradient dispersion or explosion and network degradation caused by network deep stacking, Kaiming He et al. proposed a new network structure, namely, residual network (ResNet), which constructs a new deep network by introducing a residual block [46,47]. The essence of the ResNet design is to ensure that the internal structure of the model has the ability of identity mapping so that the deep network has the same performance as the shallow network. Through identity mapping, there is no degradation due to continued stacking in the process of stacking the network. The block structure of the Identity Residual module (Identity_RES) is shown in Figure 9.

The output H(x) in model is:

(6) H(x)=F(x)+x

where *F*(*x*) is the residual mapping after learning, *H*(*x*) is the low-level mapping of the partial fitting, and *x* is the input vector. Usually, *F*(*x*) is expressed as $F(x,\left\{W_{n}\right\})$ to highlight the relationship between the input weights and update weights. Therefore, the *n*-th residual unit can be expressed as

(7) $$y_{n}=h(x_{n})=F(x,\left\{W_{n}\right\})$$

**Figure 10:**
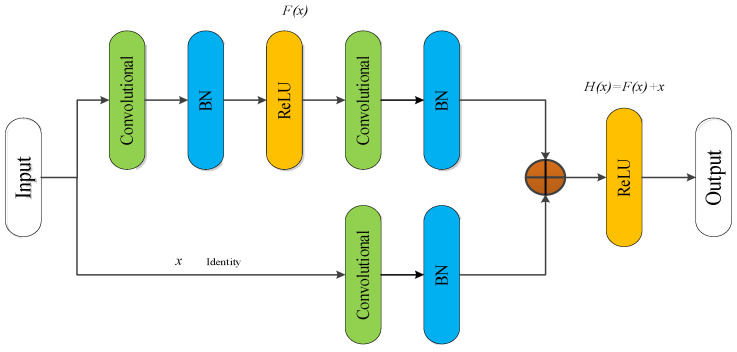
Convolutional residual module.

(8) xn+1=fReLU(yn)

where $x_{n+1}$ and $x_{n}$ represent the output and input of the *n*-th residual unit, respectively. $h(x_{n})$ represents unit mapping, and fReLU is the ReLU activation function. As can be seen from Equation (9), the features for learning from layer *n* to layer *N* are

(9) $$x_{N}=x_{n}+\sum_{i=n}^{N-1}F(x_{i},W_{i})$$

(10) $$\frac{\partial loss}{\partial x_{n}}=\frac{\partial loss}{\partial x_{N}}\cdot \frac{\partial x_{N}}{\partial x_{n}}=\frac{\partial loss}{\partial x_{N}}\left(1+\frac{\partial }{\partial x_{N}}\sum_{i=n}^{N-1}F(x_{i},W_{i})\right)$$

where Equation (10) represents the actual updated gradient of the loss when passing through the *n*-th layer. The first part in the formula represents the preserved gradient of directly transmitting the original features through the identity channel. The second part is the residual gradient related to the weight parameters of the residual network. If the output size of the previous layer and the input size of the current layer do not match each other, it is necessary to add a convolutional layer to match the output of the previous layer, that is, the Convolutional Residual module (Conv_RES). The structure of the convolutional residual module is shown as in Figure 10. Where the convolution kernel size on the identity is set to 1 × 1, the stride is set to 1 × 2, and the number of convolution kernels N depends on the specific structure.

#### 2.3.2. MFL In-Line Inspection Signal Identification Based on Improved Deep Residual Network

In this paper, an MFL signal identification method based on improved residual convolutional neural network is proposed, in which a new network is constructed by improving the residual network and introducing an attention module. The feature identification model proposed in this method has the following advantages: (1) A convolution network is adopted to extract features from the original data, which reduces the difficulty of feature extraction and enhances the universal applicability of pipeline defects and pipeline features. (2) The attention layer is added to obtain the weighted feature map under the joint action of channel attention and spatial attention to further extract the image feature information and reduce the noises impact on the feature identification results during the inspection in the pipeline. (3) Two different residual modules (identity and convolution) are introduced to deepen the depth of the deep network but to effectively reduce the computing number of parameters, decrease the errors of the deep network, save the training time, and improve the training effect.

**Figure 11:**
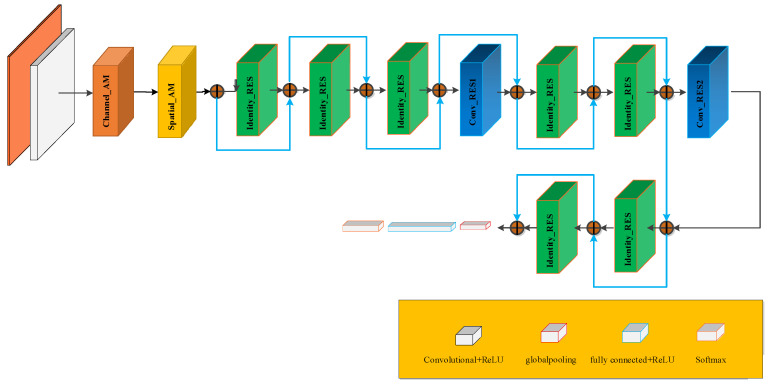
Improved deep residual network based on VGG16.

The improved residual convolutional neural network model based on VGG16 proposed in this method is illustrated in Figure 11. The input of the entire network is a pseudo-color image of the MFL in-line inspection. After data preprocessing, the image size was set to 112 × 112 × 3. After normalization, the RGB value is converted to the range of (0, 1). The normalized image is input to the first convolutional layer which has sixteen 3 × 3 convolution kernels with a stride of 1, then is input into Channel_AM and Spatial_AM after passing through the batch standardization layer and activation layer. It then enters three consecutive identical residual modules, each of which includes 16 convolution kernels with a size of 3 × 3 and a stride of 1, and has an output of 112 × 112 × 16. Next, the feature map is fed into the convolutional residual module, and on the one hand, the feature map A is obtained through ReLU activation function → convolution → batch standardization → ReLU activation function → convolution → batch standardization, on the other hand, feature map B is obtained after convolution → batch standardization. More features can be extracted from feature maps A and B through the superposition of the merging layer, and the size of the output feature map was 56 × 56 × 32. Then, the feature map passes through two identical residual modules, one convolutional residual module, and two identical residual modules, and an output image of 28 × 28 × 64 is obtained. The obtained feature map was fed into the global mean pooling layer to reduce the number of parameters and overfitting. Finally, it was connected to the full connection layer using Softmax for classification. The specific parameters for the feature identification and classification of the actual network structure are listed in Table 1.

## 3. Experiment and Results Analysis

### 3.1. Establishment of Pipeline MFL Feature Image and Sample Set

**Figure 12:**
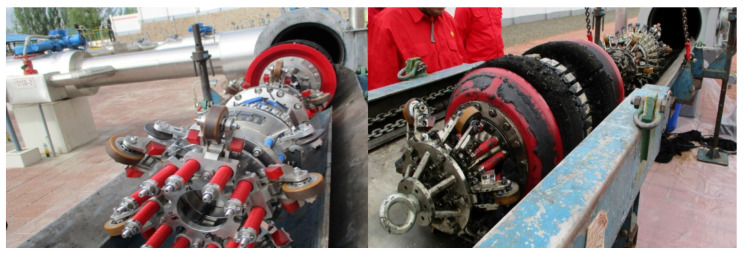
Status of in-line tool before and after running.

An in-line inspection tool is driven by the pipeline transport medium (crude oil or natural gas) to run in the pipeline, inspect, and locate the deformation, corrosion, and other damages to the pipeline in real time. Most oil and gas pipelines are buried underground. Through in-line inspection of the pipeline, various defects and damages can be found in advance, and the risk degree of each pipeline section can be understood to prevent and effectively reduce accidents and save pipeline maintenance funds, which is an important method to ensure pipeline safety. The status of in-line tool before and after running are shown in Figure 12. It can be seen from Figure 12, that there are wax, dirt, and other impurities in the pipeline, which affect the quality of the long-term signal acquisition of the tool. In addition, the tool is susceptible to welds and other influences, resulting in the lift-off of the sensor and other phenomena, further making the inspection signals vulnerable to noise interference.

**Figure 13:**
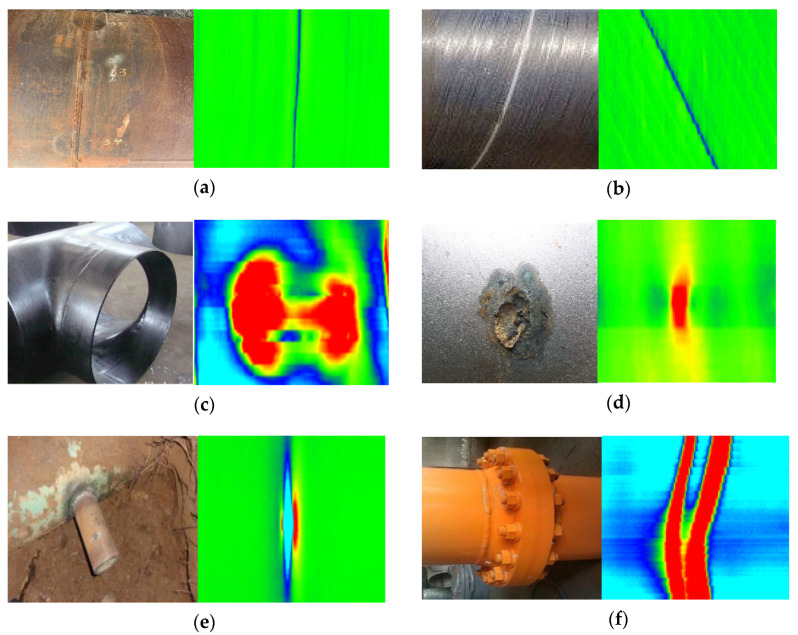
MFL pseudo-color images of different pipeline features (**a**) Girth weld. (**b**) Spiral weld. (**c**) Tee. (**d**) Corrosion. (**e**) Illegal hot tapping. (**f**) Flange.

During the inspection, the tool collects MFL signals with obvious features when passing by pipeline accessories and defects, such as welds, tees, valves, and metal loss or increase. Identifying the type of pipeline accessories according to the features of the signals, recording their locations, and other relevant information are important for MFL inspection signal analysis. Figure 13 shows MFL pseudo-color feature images of six types of pipeline defects, namely, Girth Welds (GW), Spiral Welds (SW), Tees (Tee), metal Corrosion (COR), Illegal Hot Tapping (IHT), and Flanges (FL). GW is the weld that joints the two pipes together. The GWs are orthogonal to the pipeline centerline in MFL signal. SW is a weld obtained by welding a coiled sheet of material into a tubular part in a spiral manner. In the MFL signal, it can be seen that the SW has an angle with the centerline of the pipeline. Tee can be regarded as a large metal loss signal in the MFL signal, and the outer contour is circular. Different sizes of Tees show different sizes of metal loss signals on MFL images. COR includes two types of pitting defects and extensive corrosion. Due to the different depths and sizes of pipeline corrosion defects, the leakage magnetic fields inspected by the in-line inspection tool are different. As a result, different corrosion defects have different expressions on the magnetic flux leakage image. IHT means that criminals have damaged the pipeline in order to steal oil and gas, resulting in a through hole defect in the pipeline. It can be seen from the MFL signal that the middle part is the metal loss signal. Due to the external valve, the metal increase signal is also generated. FLs are another way of connecting pipelines, in addition to the two weld signals, there is a larger metal increase signal in the middle. These six types of MFL signals can be collected by the sensors of the in-line inspection tool. However, due to different pipeline sizes, the tool operates in a complex environment inside the pipeline and other factors, the same type and different sizes of defects have different performances in the MFL images. Especially for the three signals of Tee, COR, and IHT which all contain metal loss characteristics, and identification of small and medium corrosion defect, the accurate identifying and judging is challenging. Efficient, accurate, and automatic identification can significantly improve the efficiency of MFL data analysis.

The training process of a deep convolutional neural network for target image identification and classification is a supervised neural-network training method. The input of the CNN is MFL images, and the feature types of the MFL images must be defined manually. Therefore, it is necessary to create experimental datasets and label files for various feature types. The datasets contain features images of various pipelines as far as possible. In the method, a database of MFL pseudo-color images was established, including six categories (GW, SW, Tee, COR, IHT, and FL). The dataset contains a total of 9000 pipeline MFL pseudo-color images, in which 6000 images were randomly selected as the training set, with 1000 images for each of the six categories features and defects, and the remaining 3000 images as the testing set, with 500 images for each of the six categories features and defects. The image size was set as 112 × 112 × 3.

### 3.2. Selection of Hyperparameters

The purpose of convolutional neural network training is to minimize the final loss. The final loss function adopted by the model is a cross-entropy function expressed as follows:

(11) $$J(\omega ,b)=\frac{1}{m}\left(\sum_{i=1}^{m}\left(-y^{\left(i\right)}\mathrm{lg}{\hat{y}}^{\left(i\right)}-(1-y^{\left(i\right)})\mathrm{lg}(1-{\hat{y}}^{\left(i\right)})\right)\right)$$

where *m* represents the number of samples, $y^{\left(i\right)}$ represents the label of the *i*-th sample, and *J* represents the neural network output value of the *i*-th sample.

Batch training was conducted in the experiment, and the final parameters were selected as follows. The batch size was set as 128, and the random inactivation probability of the dropout neurons was set as 0.3, and the Adam algorithm with adaptive learning rate was selected as the optimization algorithm. The learning rate attenuation strategy was adopted for learning, for which the learning rate would be reduced by 20% when the testing accuracy was no longer improved, and the lower limit of the learning rate was set as 0.00001.

### 3.3. Neural Network Model Training

**Figure 14:**
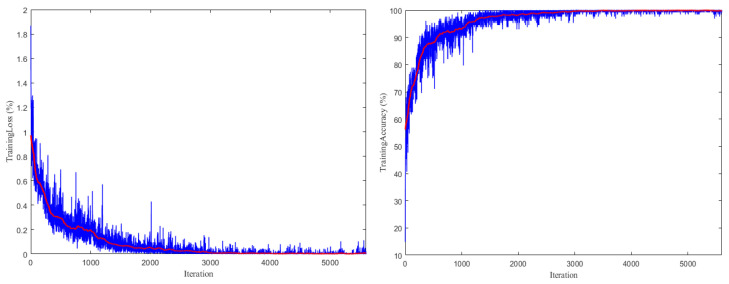
Loss and accuracy for the model.

Network training was conducted for 80 cycles, totaling 5600 iterations for training. The curve of the model loss value changing with the number of iterations in the training process and the prediction accuracy curve of the training and testing changing with the number of iterations are shown in Figure 14.

The experimental results showed that the loss value of the model decreased rapidly and the accuracy increased rapidly in the first 2000 training sessions. At approximately the 3000th training session, the model began to converge, and finally when the training reached 3500 iterations, the loss value of the model tended to be stable. It can be seen that the final parameter setting was appropriate, and the accuracy rate and loss value change trends were good.

### 3.4. Model Performance Evaluation

**Figure 15:**
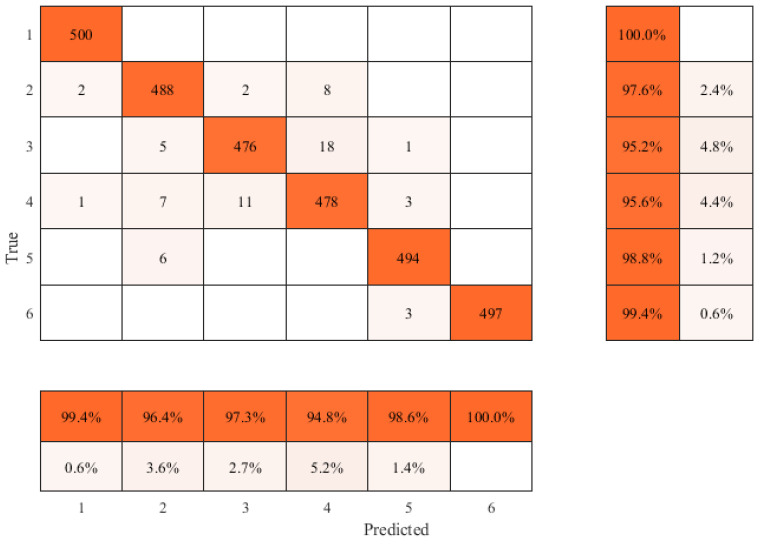
Confusion matrix for the classification.

The confusion matrix of the classification results obtained using the proposed method is shown in Figure 15. The performance of the trained neural network model was evaluated by classification accuracy of testing set, and the following evaluation indexes were used:

(1) Recall: This is the proportion of the correctly predicted samples of a certain category to the total positive samples number of this category in the testing set, reflecting the ability of the model to find defects.

(12) $$\mathrm{recall}=\frac{\mathrm{TP}}{\mathrm{TP}+\mathrm{FN}}$$

(2) Precision (PRE): This is the proportion of true samples in the samples of predicted as positive within a certain category of testing samples, reflecting the accuracy of the testing. The calculation formula is as follows:

(13) $$\mathrm{Precison}=\frac{\mathrm{TP}}{\mathrm{TP}+\mathrm{FP}}$$

(3) Accuracy (ACC): The accuracy is used to measure the proportion of the number of correctly predicted samples to the total number of samples. The higher the accuracy, the better the performance of the model. The calculation formula is as follows:

(14) $$\mathrm{Accuracy}=\frac{\mathrm{TP}+\mathrm{TN}}{\mathrm{TP}+\mathrm{TN}+\mathrm{FP}+\mathrm{FN}}$$

True Positive (TP) is the number of positive examples that are correctly divided. False Positive (FP) is the number of false positives that are incorrectly divided. False Negative (FN) is the number of negative examples that are incorrectly divided. True Negative (TN) is the number of negative examples that are correctly divided.

As shown in Figure 15 and Table 2, the improved residual convolutional neural network model based on VGG16 proposed in this method can achieve 97.7% identification accuracy for six types of features. The recall and accuracy rates of GW are 100% and 99.4%, respectively; and the recall and accuracy rates of SW are 97.6% and 96.4%, respectively; the recall and accuracy rates of Tee are 95.2% and 97.3%, respectively, the recall and accuracy rates of COR are 95.6% and 94.8%, respectively; the recall and accuracy rates of the IHT are 98.8% and 98.6%, respectively; and the recall and accuracy rates of FL are 99.4% and 100%, respectively.

**Figure 16:**
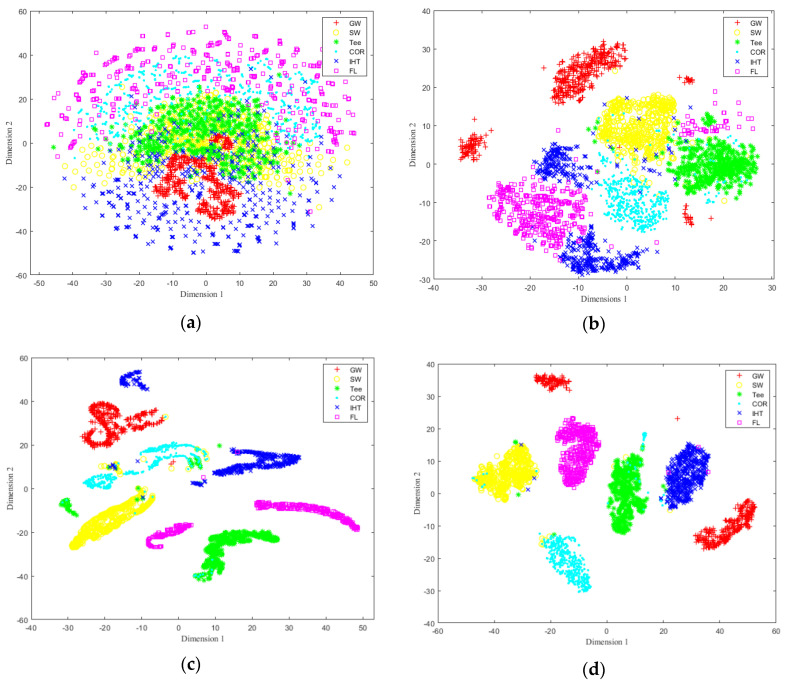
T-SNE of feature identification and classification at different stages. (**a**) Original training set. (**b**) Classification results after Conv_RES1 + ReLU. (**c**) Classification results after Conv_RES2 + ReLU. (**d**) Final classification results.

To further illustrate the accuracy of feature identification for the constructed network, t-distributed stochastic neighbor embedding (t-SNE) [48] was used to reduce the dimensions of the test dataset, realize the visualization in two dimensions, and observe the distribution of sample data to determine whether it is possible to be classified. The selected training sample dataset was reduced to two dimensions by t-SNE method, and a two-dimensional distribution map of the training samples was obtained, as shown in Figure 16a. It can be found that the training sample set has certain clustering and has the possibility of being classified, and the data distribution is found to be nonlinear to a certain extent, which also poses a challenge to the nonlinear mapping ability of the classification model. Figure 16b shows the classification effect of testing sample set by the first Conv_RES1 + ReLU module, and it can be seen that by three consecutive Identity_RES + ReLU modules and one Conv_RES1 + ReLU module, the testing data set has a certain classification trend. Figure 16c shows the classification and identification effect of testing the sample set by the second Conv_RES2 + ReLU module, and it can be seen by two consecutive Identity_RES + ReLU modules and one Conv_RES2 + ReLU module, that the testing data set has been effectively aggregated and classified; Figure 16d shows the final classification and identification results of the testing sample set by passing Softmax, and the overall feature identification accuracy is 97.7%. The main reason for the classification errors is that the MFL image features were not obvious for some small oil illegal hot tappings which were misidentified as general corrosion defects, and some large metal corrosion defects were mistakenly identified as illegal hot tappings.

### 3.5. Comparisons with Other Methods

**Figure 17:**
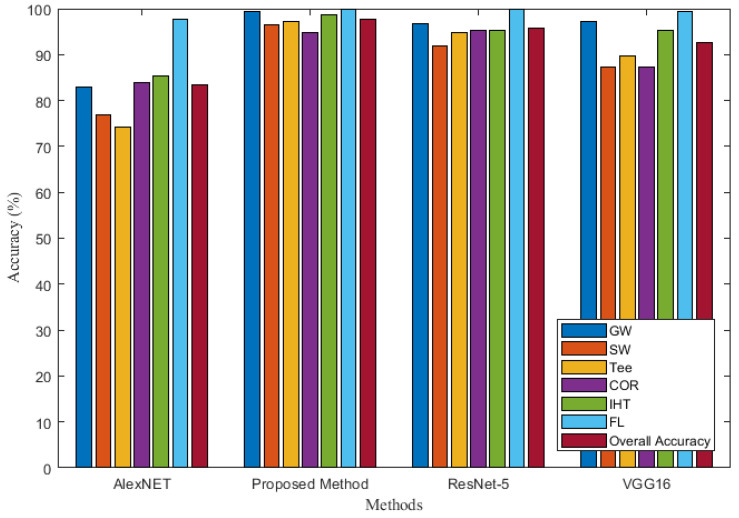
Accuracy comparisons with different methods.

To test and verify the performance of the proposed improved residual convolutional neural network based on VGG16, three methods of deep CNN—AlexNET [49], ResNet-50 [50], and VGG16—were selected for comparison with the proposed method. Comparison results of the four methods is presented in Table 3 and Figure 17. As shown in Table 3 and Figure 17, when the identification is based on Alexnet network, the PRE and recall of SW and Tee reaches lower than 80%. When the identification is performed using the VGG16 network, the PRE and recall of six types corrosion defects MFL signals all improved and reaches higher than 80%. When the identification based on ResNet-50 network, the PRE of GW defect reaches 96.7%, the PRE of SW defect reaches 91.9%, the PRE of Tee defect reaches 94.9%, the PRE of COR defect reaches 95.2%, the PRE of IHT is 95.3% and the PRE of FL is 100%. The PRE of six types corrosion defects by the proposed method are 99.4%, 96.4%, 97.3%, 94.8%, 98.6%, and 100%, respectively. The recall of the six types of corrosion defects by the proposed method are 100%, 97.6%, 95.2%, 95.6%, 98.8%, and 99.4%, respectively. The PRE and recall by the proposed method are the highest compared with the previous three methods. A comparison of the results of the above four identification methods reveals that the proposed method has the highest identification accuracy for the GW, SW, Tee, and IHT defect. It can be found that for the MFL features of a single pipeline, the identification accuracy of COR and IHT is low because the two signal features are similar, especially when the IHT has a smaller size, COR has a larger size, or the external branch pipe feature of IHT is not obvious, misjudgment often occurs. The identification accuracy of FL is the highest because FL signals are the easiest to identify compared to other feature signals.

It can be seen that the average identification accuracies of AlexNET, VGG16, ResNet-50, and the proposed method are 83.5%, 92.67%, 95.7%, and 97.7%, respectively. The analysis of four deep convolutional neural networks shows that the identification accuracy of ResNet-50 and the proposed method are higher than those of AlexNET and VGG16, and the proposed method obtained the best classification results.

**Figure 18:**
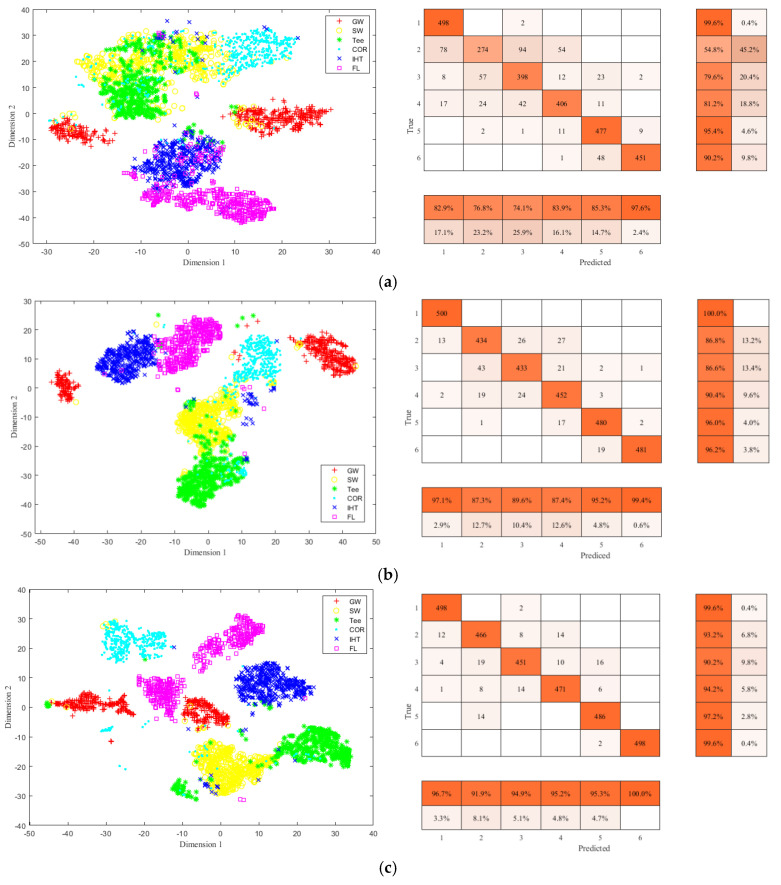
The visualization results and confusion matrix of different methods. (**a**) AlexNET. (**b**) VGG16 NET. (**c**) ResNet-50.

To more intuitively show the feature classification accuracy of different methods, t-SNE and confusion matrix are used to show different classification results. Figure 18 shows the classification results of the pipeline features using AlexNET, VGG16 NET, and ResNet-50. From Figure 18a it can be seen that some of GW, SW, and Tee samples are confused, and IHT and FL samples are not well separated. In Figure 18b,c, the classification effects improve and most samples are effectively separated. From the classified features distribution by the proposed method shown in Figure 16d, it can be seen that by the proposed method the six types of pipeline features can be effectively separated and each type of pipeline feature sample clusters well.

In the proposed method the attention layer is introduced to obtain the channel attention module, which can fully extract the MFL image feature information and reduce the influence of noises on the feature identification results during the pipeline inspection. Two different residual modules (identity and convolution modules) are introduced to deepen the depth of the deep network, effectively reducing the number of computing parameters, reducing the errors of the deep network, and effectively preventing the gradient dispersion or explosion and network degradation caused by network deep stacking. In addition, different residual modules can better model MFL features for pipeline defects, the stacked deep residual network can perceive more detailed information contributing to the identification of pipeline features, and effectively improve the identification accuracy.

## 4. Conclusions

Long-distance oil and gas pipelines are an important lifeline of the national economy. During long-term service and operation, the pipeline will be affected by corrosion and third-party construction, resulting in pipeline leakage or explosion, which poses a great threat to property, life safety, and the natural environment. Periodic inspection and maintenance of pipelines using MFL in-line inspection and other methods are important for maintaining the integrity of the pipeline. The rapid and accurate identification and analysis of pipeline MFL in-line inspection data can improve the efficiency of pipeline maintenance. However, during in-line inspection for girth welds and spiral welds, the difference in weld direction is likely to cause a decrease in the identification accuracy. For defects, such as corrosion and hot-tappings, due to the influence of sensor lift-off, the difference in defect size, the influence of noise during motion, and the compound features, it is difficult to effectively identify and classify different defects and features inspected by MFL. To improve pipeline MFL signal identification and classification accuracy, a pipeline MFL inspection signal identification method based on improved deep residual convolutional neural network is proposed. The main contents and innovations of this study are as follows:

(1) In view of the influence of complex operating environments, high noises, compound defects, and features on the feature identification results in the pipeline, the attention module method is constructed. The channel attention and spatial attention structures are adopted to fully extract the image feature information to improve the classification accuracy.
(2) Aiming at the problem that the gradient dispersion caused by the increase in network layers makes it difficult for the model to converge and network degradation, an improved residual convolutional neural network based on VGG16 is constructed. The identity and convolution residual modules are introduced to reduce the errors of the deep network and the number of computing parameters, and improves the training efficiency.
(3) By field test, the pipeline feature types were analyzed and sample sets of the MFL pseudo-color images were established. Experiment results indicate that the identification accuracy of six types of pipeline features and defects reached 97.7%.

This paper presents a pipeline MFL inspection signal identification method based on improved deep residual convolutional neural network. The special attention linked deep residual network is constructed to optimize the VGG16 and attention modules are embedded. The improved network can extract exact MFL features, and effectively prevent gradient dispersion due to more network layers. The application of this method greatly improves the accuracy and efficiency of identification for MFL features and defects, and provides methods for the safe operation of oil and gas pipelines.

## Figures and Tables

**Table 1:** Structure and parameters of the improved deep residual network based on VGG16.

Layer Number	Network	Kernel Size	Stride	Kernel Number	Output	Other
1	Input				112 × 112 × 3	
2–4	Conv + BN + ReLU	3 × 3	1 × 1	16	112 × 112 × 16	Same
5–15	Channel_AM				112 × 112 × 16	
16–22	Spatial_AM				112 × 112 × 16	
23–43	(Identity_RES + ReLU) × 3				112 × 112 × 16	
44–52	Conv_RES1 + ReLU				56 × 56 × 32	
53–66	(Identity_RES + ReLU) × 2				56 × 56 × 32	
67–75	Conv_RES2 + ReLU				56 × 56 × 32	
76–89	(Identity_RES + ReLU) × 2				28 × 28 × 64	
90	POOL	8 × 8			21 × 21 × 64	Global
91	FC				1 × 1 × 6	
92	Softmax				1 × 1 × 6	
93	Classification				1 × 1 × 6	

**Table 2:** Performance of the proposed method.

	GW	SW	Tee	COR	IHT	FL
Recall	100%	97.6	95.2%	95.6%	98.8%	99.4%
PRE	99.4%	96.4%	97.3%	94.8%	98.6%	100%
ACC	97.7%					

**Table 3:** Accuracy comparison of different methods.

Identification Model	Index	GW	SW	Tee	COR	IHT	FL	Number of Network Layers
AlexNET	Recall	99.6%	54.8%	79.6%	81.2%	95.4%	90.2%	8
	PRE	82.9%	76.8%	74.1%	83.9%	85.3%	97.6%	
	ACC	83.5%						
VGG16	Recall	100%	86.8%	86.6%	90.4%	96%	96.2%	41
	PRE	97.1%	87.3%	89.6%	87.4%	95.2%	99.4%	
	ACC	92.67%						
ResNet-50	Recall	99.6%	93.2%	90.2%	94.2%	97.2%	99.6%	50
	PRE	96.7%	91.9%	94.9%	95.2%	95.3%	100%	
	ACC	95.7%						
The proposed method	Recall	100%	97.6%	95.2%	95.6%	98.8%	99.4%	93
	PRE	99.4%	96.4%	97.3%	94.8%	98.6%	100%	
	ACC	97.7%						

## Data Availability

The data presented in this study are available from the corresponding author upon reasonable request.
